# Aerobic exercise and NBS superfood supplementation modulate iron-related biomarkers in methadone treated men in randomized double blind controlled trial

**DOI:** 10.1038/s41598-025-32518-5

**Published:** 2025-12-18

**Authors:** Fatemeh OmidAli, Negin Kordi, Aref Khalkhali

**Affiliations:** 1 Department of Sport Sciences, Faculty of Humanities, Ayatollah Ozma Boroujerdi University, Boroujerd, Iran; 2 Department of Exercise Physiology, Faculty of Sport Sciences, Razi University, Kermanshah, Iran; 3 Department of Science, Faculty of Biology, Islamic Azad University, Mashhad, Iran

**Keywords:** SLC7A11, GPX4, Hepcidin, Ferritin, Ferroptosis, Addiction, Biochemistry, Biomarkers, Diseases, Medical research

## Abstract

Methadone maintenance therapy effectively treats opioid addiction but disrupts iron metabolism and promotes ferroptosis. This study aimed to investigate the combined effects of aerobic exercise and NBS superfood supplementation on iron-related biomarkers in men undergoing methadone maintenance therapy. Fifty-eight participants were randomized into four groups: control, exercise, supplement, and exercise + supplement. The 8-week aerobic exercise protocol targeted 65% of peak heart rate. The supplement group received 4.5 g/day of NBS. Serum levels of GPX4, SLC7A11, hepcidin, and ferritin were measured via ELISA. Data were analyzed using repeated measures ANOVA in SPSS-26. Significant time × group interactions were observed for GPX4, SLC7A11, and ferritin (*p* < 0.05), with the largest reductions seen in the combined exercise + supplement group. Hepcidin levels did not change significantly in any group (*p* > 0.05). The combined intervention showed the greatest improvement in these biomarkers, though the magnitude of changes was moderate. Eight weeks of aerobic exercise combined with NBS superfood supplementation was associated with favorable changes in GPX4, SLC7A11, and ferritin levels in men undergoing Methadone maintenance therapy, suggesting a possible modulation of iron-related pathways. However, the clinical significance of these biochemical changes remains to be established. The study is limited by its relatively small sample size, male-only population, short duration, and lack of long-term follow-up. Larger and longer-term trials are needed to confirm these preliminary findings and to assess whether the observed biomarker changes translate into meaningful clinical benefits.

## Introduction

Drug addiction is a major public health challenge worldwide, with widespread physical complications in addition to negative mental health impacts^1^. As reported by the United Nations Office on Drugs and Crime, the global population engaging in drug use reached approximately 296 million individuals in 2022, reflecting a 20% rise compared to the preceding decade. This escalation imposes a significant strain on healthcare systems worldwide^2^. Methadone maintenance treatment is an effective drug treatment for opioid addiction that helps individuals maintain a relatively stable level of opioids in their bodies^3^. Although methadone is effective in reducing withdrawal symptoms and improving quality of life, Halcrow et al. (2022) reported that opioid drugs such as methadone, by disrupting Fe^2+^ metabolism, can lead to mitochondrial dysfunction and iron-dependent cell death^4^. Ferroptosis is a type of iron-dependent programmed cell death that is genetically regulated by several genes^4^. The xC-GSH-glutathione peroxidase 4 (GPX4) system plays a crucial role in regulating ferroptosis^5^. GPX4 converts intracellular lipid hydrogen peroxide to lipid alcohol, enhances hydrogen peroxide degradation, repairs oxidative damage to lipid cells, and protects cell membranes from oxidative damage. Inactivation of GPX4 leads to the accumulation of lipid peroxides and ferroptosis^6^. The cystine/glutamate antiporter (system xc⁻), composed of the catalytic subunit SLC7A11 (xCT) and the regulatory subunit SLC3A2 (4F2hc), facilitates cystine uptake in exchange for glutamate, thereby supplying cysteine for glutathione synthesis and maintaining redox homeostasis in ferroptosis-sensitive cells^7^. These subunits synthesize glutathione (GSH) and scavenge intracellular free radicals through the exchange of intracellular glutamate and extracellular cysteine. Suppression of the xc⁻system can lead to GSH depletion and ferroptosis^8^. A study by Kosari et al. (2025) demonstrated that methadone administration in rats resulted in elevated levels of the antioxidant enzymes glutathione peroxidase (GPx) and catalase, alongside increased malondialdehyde (MDA), a marker of oxidative stress, in renal tissue^9^. Therefore, based on this mechanistic evidence, it is hypothesized that methadone therapy might create a cellular environment conducive to ferroptosis, highlighting a potential novel mechanism for its side effects and a target for protective interventions.

Specifically, methadone may increase hepcidin expression by activating the IL-6/STAT3 inflammatory pathway, which binds to ferroportin, stimulating its degradation in lysosomes and preventing iron release from cells^10^. Ferritin, a major indicator of body iron stores, is also stored in cells (especially the liver and macrophages) and plays a key role in protecting cells against oxidative stress caused by free iron. The accumulation of iron in cells (caused by ferroportin inhibition) increases oxidative stress through the production of free radicals and activates the ferroptosis pathway^11^.

Recently, non-pharmacological interventions such as exercise training and the use of nutritional supplements have been considered as strategies to regulate body homeostasis in different individuals. A study by Domínguez (2018) shows that aerobic exercise can reduce hepcidin levels^12^. Aerobic exercise may increase SLC7A11 expression and GPX4 activity, which may contribute to improved antioxidant defenses^13^. Šmid et al. (2024) also showed that the combination of aerobic exercise and iron supplementation in some studies resulted in improved ferritin levels, indicating better iron balance under exercise conditions^14^.

On the other hand, the use of dietary supplements and herbal medicines has increased significantly in recent decades^15^. One of the main concerns of consumers of herbal supplements is the lack of sufficient knowledge about the ingredients and side effects of supplements. Research on dietary supplements shows that their ingredients, such as vitamins, sterols, dietary fibers, flavonoids, and other antioxidant compounds, can help improve systemic and metabolic disorders^16^. Antioxidant molecules found in wheat germ and grains have been shown to prevent oxidative damage to deoxyribonucleic acid (DNA) in vitro^17^. Wheat is a rich source of phytosterols, policosanol, tocopherols, thiamine, niacin, and riboflavin^18^and, as a rich dietary source of non-heme iron, plays an important role in regulating ferritin (an iron-storing protein). Studies show that whole wheat consumption increases iron absorption and improves serum ferritin levels^19^.

Nutrition Bio-Shield (NBS) is an organic herbal supplement derived from wheat germ. It is a natural product rich in essential micronutrients, including a comprehensive profile of vitamins (such as thiamine, riboflavin, niacin, vitamins E, C, and K), mineral elements (including phosphorus, iron, copper, and selenium), and bioactive compounds like flavonoids^20^. Notably, its content of vitamin E and selenium (key structural components for the antioxidant enzyme GPX4) underscores its potential role in supporting critical antioxidant pathways in the body^21^. These compounds may have protective effects against oxidative stress caused by iron accumulation by supporting the SLC7A11-GPX4 system. To date, no published studies have examined the effects of NBS superfood supplementation in men receiving methadone maintenance therapy. The main question of this study is whether the combination of aerobic exercise and consumption of the NBS superfood can restore iron balance in this population by changing the activity levels of SCL7A11 and GPX4 and regulating ferritin stores. This study is one of the few studies that simultaneously examines four key indicators of iron metabolism in methadone users and can provide a basis for the design of combined “exercise-nutrition” protocols to reduce the metabolic complications caused by methadone use. Therefore, this study was designed to investigate the combined effect of aerobic exercise and NBS superfood supplement consumption on the levels of SCL7A11, GPX4, hepcidin, and ferritin in men using methadone.

## Materials and methods

### Participants

The sample size was calculated using G Power software in conjunction with the F test from the repeated measures ANOVA, leading to a total of 56 individuals. The type I error rate was established at 0.05, with a minimum effect size of 0.40; the number of groups was set at 4, two measurements, and the Statistical power was 0.80^22^.

To account for potential participant attrition, the final sample size was determined to be 60 individuals. A randomized selection of 60 men recovering from addiction and receiving methadone maintenance therapy was recruited from the Tolo Novin Omid Center in Boroujerd, Lorestan, Iran. This study was conducted as a double-blind trial, ensuring that neither the participants nor the researchers knew who received the supplement or the placebo. Both the supplement and placebo were packaged identically (matching in appearance, labeling, and administration method) and were distributed by an independent team at the addiction treatment center, who had no involvement in other aspects of the study. The participants were randomly allocated into four equal groups (*n* = 15 per group) through simple randomization using a lottery method. This allocation process was performed by independent center staff who were not otherwise involved in the study. The participants were randomly assigned to one of the following four groups:

1. **CONT**: Control Group (Received a placebo only).
2. **SUP**: Supplement Group (Received the supplement only).
3. **TR**: Training Group (Combined exercise training with placebo intake).
4. **TR + SUP**: Training + Supplement Group (Combined exercise training with supplement intake).

The research was conducted as a double-blind trial, ensuring that neither the participants nor the investigators knew the group allocations (supplement or placebo). To maintain blinding, the supplement and placebo were packaged identically in terms of appearance, labeling, and administration. An independent team from the addiction center, not involved in other study procedures, handled the distribution.

A participant from the TR group decided to withdraw, and a person from the SUP group was expelled due to a relapse into drug use.

#### Inclusion criteria

Eligible participants were required to fulfill the following criteria: holding a valid morphine test certificate, being in methadone maintenance treatment for at least three months, being aged between 25 and 35 years, showing adequate physical fitness, and having no history of autoimmune diseases, cardiovascular or respiratory issues, or musculoskeletal injuries.

#### Exclusion criteria

Participants were excluded from the study if they resumed drug use, incurred any sports-related injuries, failed to follow the exercise protocol, or opted out of taking the prescribed dietary supplements.

### Dietary protocol

All participants followed a standardized meal plan according to the camp’s nutritional guidelines. They were instructed to consume all meals provided and were strictly prohibited from ingesting any substances other than the approved supplements under close supervision.

### Methadone administration

The camp physician administered 3 cc of methadone syrup diluted in water to participants daily between 9:00 and 10:00 AM, following breakfast.

### Training protocol

Initially, participants engaged in an introductory session that included practice and testing procedures. The peak capacity of the patients was assessed during this first session to establish the training intensity. To evaluate the intensity of training based on peak power output, participants first cycled for 20 min on a 20-watt Ergoline bicycle ergometer (German SELECT 100, ECG mountable) for warm-up. Subsequently, the initial resistance was set at 60 watts, with an increment of 15 watts added each minute. In instances of voluntary exhaustion, significant abnormalities in the ECG (a drop in the ST-segment exceeding 2 mm, or an abnormal blood pressure response) and high exercise training pressure (Borg scale: 7 or above) necessitated cessation. The training regimen was designed according to the percentage of peak output. Furthermore, according to the literature, subjects randomized to the TR and TR + SUP groups commenced with 20 min of continuous activity at 65% of their peak heart rate. The control group maintained a normal lifestyle as advised by their physician but did not engage in the training program. To ensure compliance with the principle of training overload, the exercise duration was progressively increased by 5 min every two weeks. Specifically, participants performed cycling sessions for 20 min during weeks 1–2, 25 min during weeks 3–4, 30 min during weeks 5–6, and 35 min in the final two weeks (weeks 7–8).

Participants began each session with a 10-minute warm-up at 50% of their peak heart rate. In addition, each session concluded with a 10-minute cool-down at 50% peak heart rate^23^.

### Supplementation protocol

The supplementation regimen involved the oral administration of 4.5 g of NBS superfood supplement daily, divided into three equal 1.5-gram doses, administered at specific intervals: one in the morning (6:00 a.m. to 12:00 p.m.), one in the afternoon (12:00 p.m. to 6:00 p.m.), and one in the evening (6:00 p.m. to 12:00 a.m.). The powder was dissolved in 250 mL of water for each dose and consumed 30 min prior to the main meals, with the regimen sustained over eight consecutive weeks^24^. The SUP group was procured from NBS Superfood (NBS Organic Company, Turkey). The NBS food supplement has various vitamins, macro and micro molecules, and ingredients such as B1, B2, B3, B5, B6, B9, C, K, A, E, D, phosphorus, potassium, sulfur, magnesium, calcium, boron, iron, manganese, zinc, copper, omega-3, omega-6, omega-9 and, etc^25^.

Participants in both the CONT and TR groups were administered potato starch as a placebo in lieu of the NBS superfood supplement.

### Measurements

Clarifications were provided concerning the timing of blood collection and adherence to factors such as dietary habits, substance use, and smoking. All dependent variables in the study were assessed at two distinct time points (24 h before the initial training session and 24 h after the final training session). During each instance, 10 cc of blood was drawn from each participant while in a fasting state (12 h) from the brachial vein. All assessments were conducted under consistent conditions (between 8 am and 10 am). Blood samples underwent centrifugation at 10 rpm for a duration of 10 min. The resulting serum was subsequently transferred into numbered microtubes and preserved in a − 80 ◦ C freezer for future analyses. The resulting serum was subsequently transferred into numbered microtubes and stored at − 80 °C for 8 weeks until biochemical analyses were performed.

**Fig. 1:**
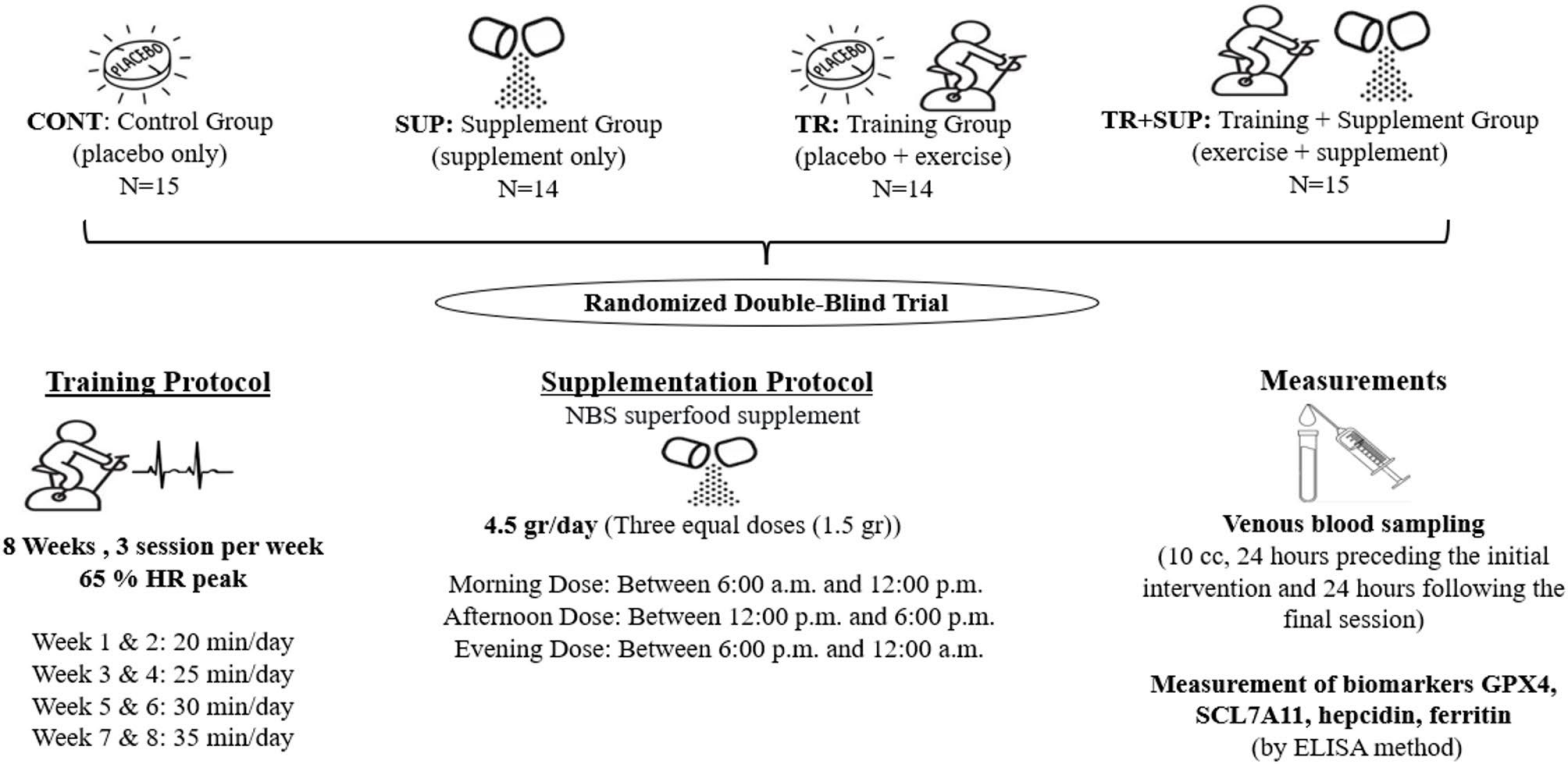
The experimental design of the study.

The following commercial assay kits were utilized for the measurements: GPX4 was quantified with an Abcam kit (United States), SCL7A11 levels were assessed using a kit from Fine Biotech Co., Ltd. (China), hepcidin was measured with a ZellBio kit (Germany), and ferritin was analyzed using a Pars Peyvand kit (Iran) (Fig. 1).

### Ethical considerations

This study received approval from the Medical Ethics Committee of the Physical Education and Sports Sciences Research Institute, Tehran, Iran (Ethics Code: IR.SSRC.REC.1404.016) and the Declaration of Helsinki. Participant confidentiality was strictly maintained throughout the study. Before participating, individuals were provided with written details regarding the study’s procedures, supplement use, and potential risks and benefits. Before the start of the study, written consent was obtained from all the participants. Participation was voluntary, and no coercion was applied. In cases of physical injury or complications, the research team provided financial and moral support until full recovery was achieved.

In this study, adverse events were defined as any physical injury (e.g., muscle strain, joint pain) or medical events (e.g., dizziness, abnormal heart rate) that occurred during or after the exercise sessions. These adverse events were assessed non-systematically through subject reports and clinical examination by the study physician. These adverse events were reported according to a monitoring protocol approved by the Ethics Committee of the Sports Science Research Institute.

Trial monitoring was conducted by the principal investigator and included checking protocol compliance, data accuracy, and recording of adverse events. External monitoring was not required due to the low-risk nature of the intervention. All methods were performed in accordance with the relevant guidelines and regulations approved by the ethics committee. The ethics committee had the right to audit the trial conduct at any time.

### Trial registration

This randomized controlled trial was prospectively registered in the Iranian Registry of Clinical Trials (IRCT) with the identifier IRCT20141228020465N7 on 17/06/2025 (First Posted), before participant enrollment. https://irct.behdasht.gov.ir/search/result?query=IRCT20141228020465N7. 

### Statistical analysis

Descriptive statistics are reported as mean ± standard deviation. Normality of data distribution was assessed using the Shapiro-Wilk test, while homogeneity of variances was verified via Levene’s test. For normally distributed data, between-group differences were analyzed using repeated measures ANOVA. Subsequent pairwise comparisons within groups were conducted using the Bonferroni post hoc test for precise determination of differences. All statistical analyses were performed in SPSS software (version 26), with the significance threshold set at *p* < 0.05. The graphs were drawn with GraphPad Prism version 8 software.

## Results

Normality of the data was checked using Kolmogorov-Smirnov’s test (*P* > 0.05), and the equality of error variance was assessed and confirmed using Levene’s test of error variance (*P* > 0.05). The general characteristics of the subjects are given in Table 1.

**Table 1:** Descriptive characteristics of the subjects.

Variables	Groups			
	CONT	Tr	SUP	TR + SUP
Age (year)	29.07 ± 3.21	28.72 ± 3.15	30.38 ± 2.45	29.56 ± 3.07
Weight (kg)	59.76 ± 2.38	58.51 ± 3.14	56.62 ± 3.90	56.23 ± 4.01
Height (cm)	173.33 ± 3.37	173.36 ± 3.45	172.64 ± 4.30	171.87 ± 3.94
BMI (kg/m^2^)	19.89 ± 0.84	19.47 ± 0.89	18.98 ± 0.84	19.03 ± 1.22
HR (beat/min)	75.60 ± 4.61	75.70 ± 4.67	74.57 ± 3.25	73.93 ± 4.55
SBP (mmHg)	9.99 ± 1.07	9.60 ± 0.69	10.29 ± 1.24	9.85 ± 0.94
DBP (mmHg)	7.14 ± 1.13	8.05 ± 0.73	8.48 ± 1.42	7.87 ± 0.85

**Fig. 2:**
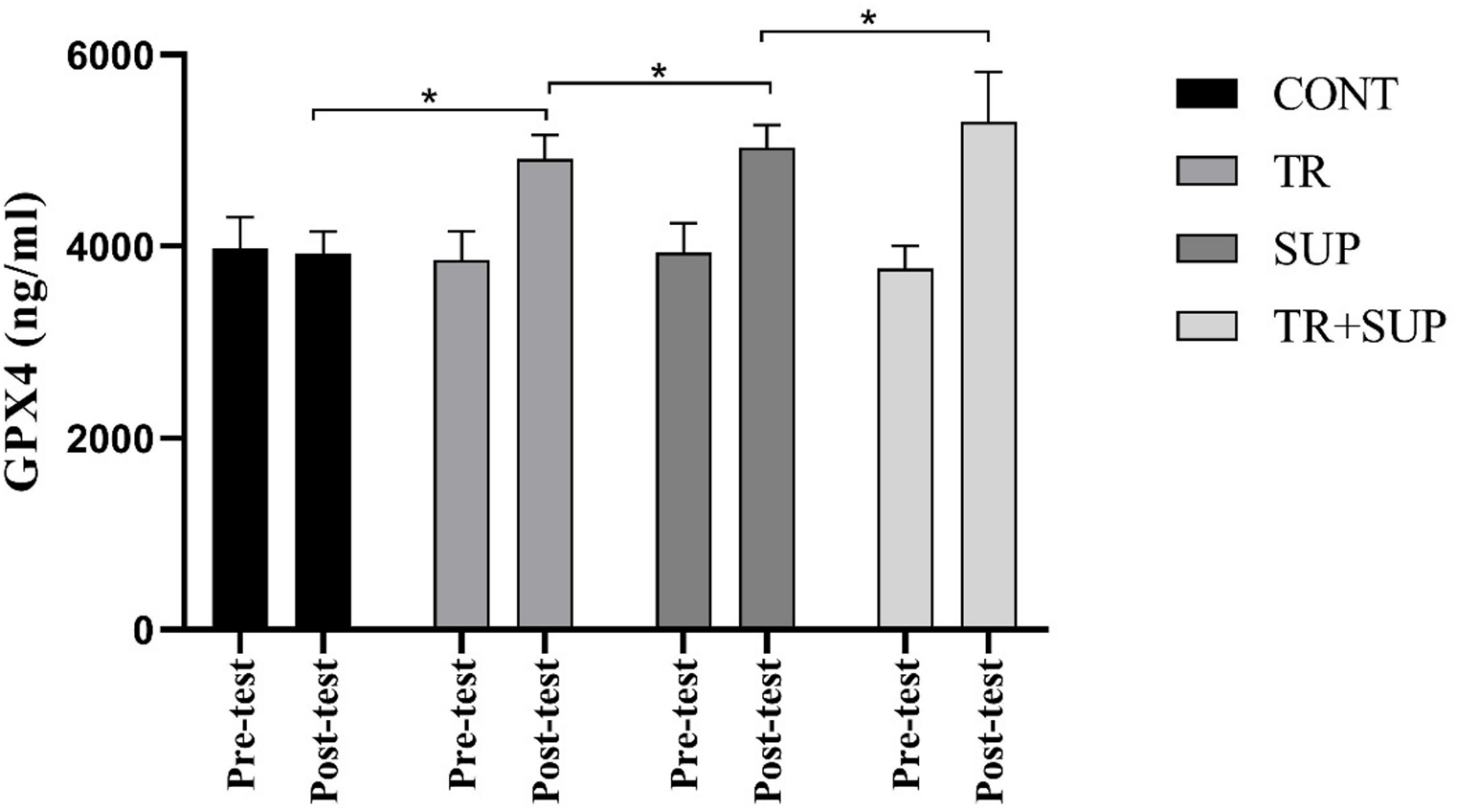
GPX4 changes levels in the three groups of CONT, TR, SUP, TR + SUP before and after 8 weeks of Interventions.

The analysis revealed a significant increase in GPX4 levels following both exercise and supplementation interventions (F = 22.83, *P* = 0.001). Post hoc comparisons showed significant differences between the CONT group and the TR group (mean difference = -522.94, *P* = 0.001), between the CONT and the SUP group (mean difference = -621.95, *P* = 0.001), and between CONT and the combined intervention TR + SUP group (mean difference = -681.82, *P* = 0.001) (Fig. 2).

**Fig. 3:**
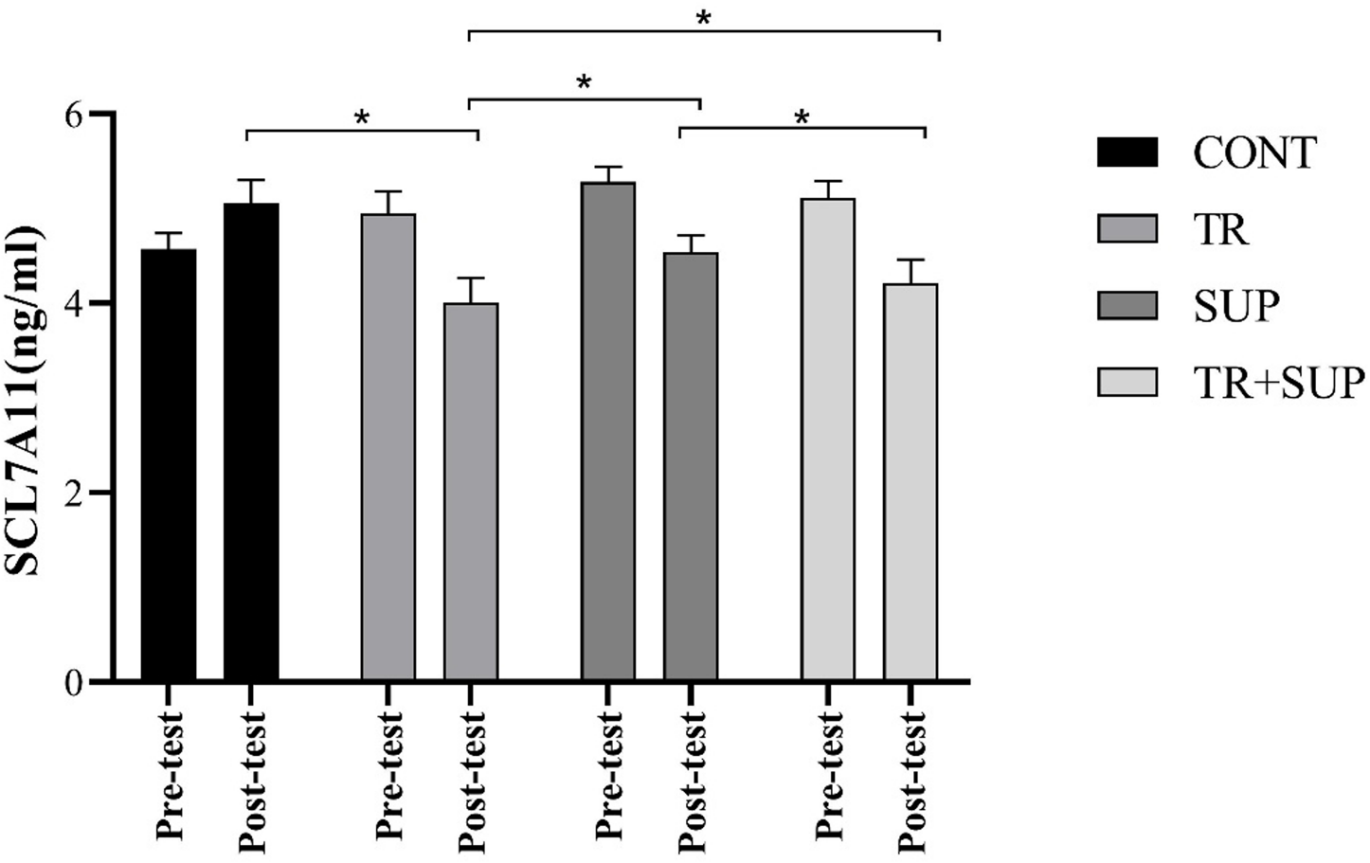
SCL7A11 changes levels in the three groups of CONT, TR, SUP, TR + SUP before and after 8 weeks of Interventions. *Significant difference between groups.

The ANOVA analysis revealed that training, supplementation, and their combination significantly decreased SCL7A11 levels (F = 15.94, *P* = 0.001). Post hoc comparisons using the Bonferroni test indicated significant differences between the CONT and TR groups (mean difference = 0.333, *P* = 0.001), the TR and SUP groups (mean difference = -0.427, *P* = 0.001), and the SUP and TR + SUP groups (mean difference = 0.252, *P* = 0.002). However, the difference between the TR and TR + SUP groups was not statistically significant (mean difference = -0.175, *P* = 0.059) (Fig. 3).

**Fig. 4:**
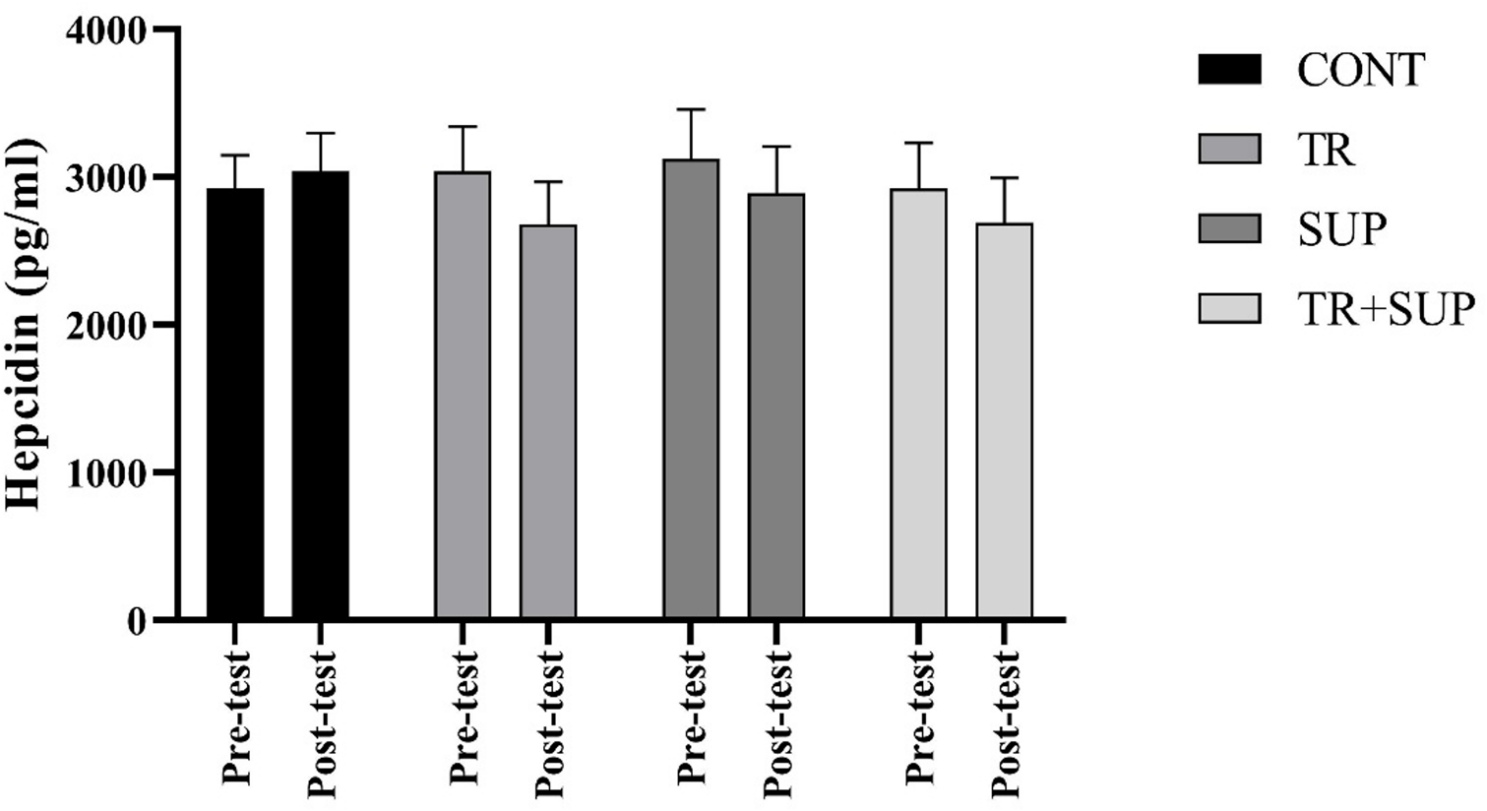
Hepcidin changes levels in the three groups of CONT, TR, SUP, and TR + SUP before and after 8 weeks of Interventions.

The analysis indicated that neither training nor supplementation had a significant effect on hepcidin levels (F = 1.72, *P* = 0.17) (Fig. 4).

**Fig. 5:**
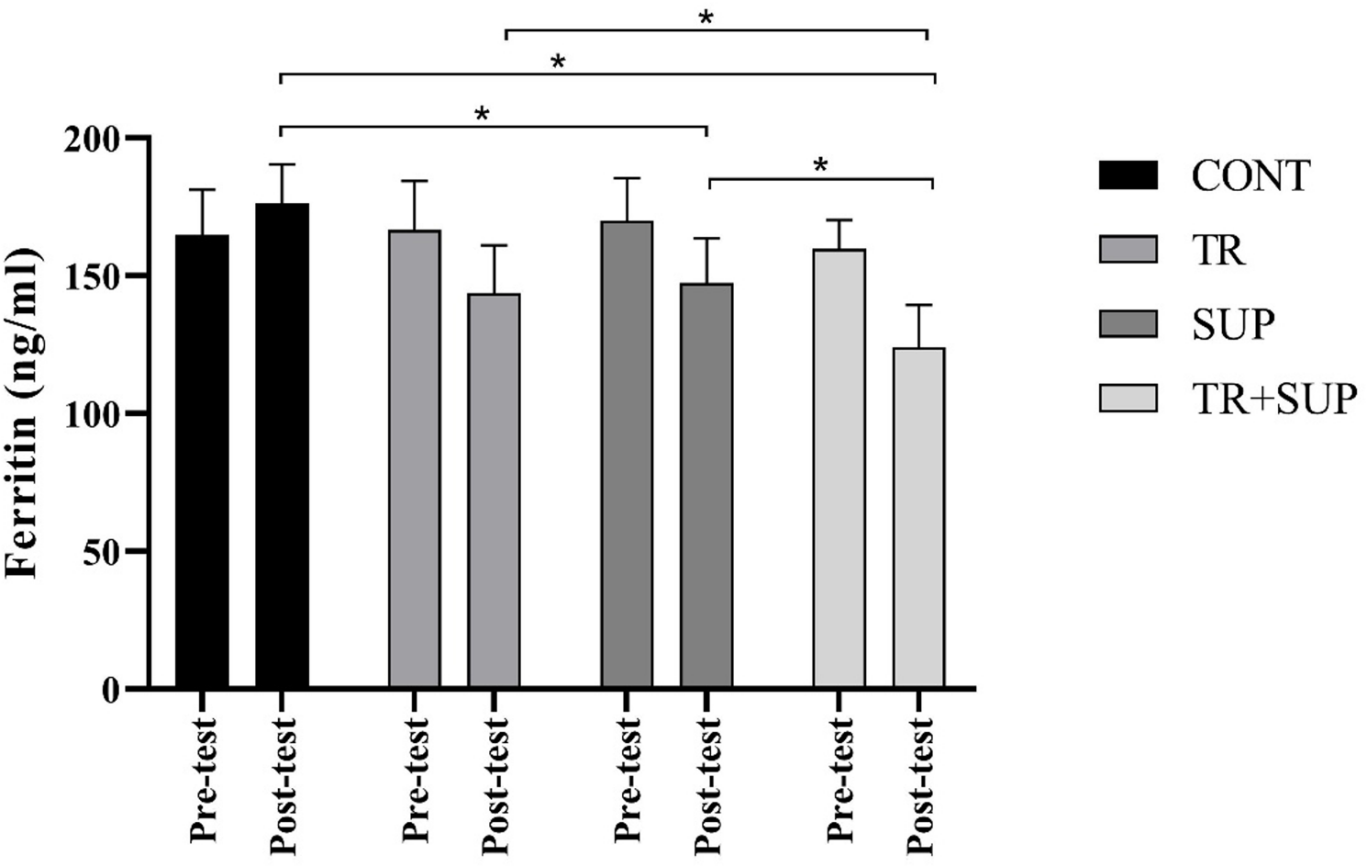
Ferritin changes levels in the three groups of CONT, TR, SUP, and TR + SUP before and after 8 weeks of Interventions.

The findings further demonstrated that both training and supplementation significantly reduced serum ferritin levels in methadone users (F = 17.92, *P* = 0.001). Post hoc analysis revealed significant differences between the CONT and TR groups (mean difference = 15.35, *P* = 0.002), the CONT and SUP groups (mean difference = 11.71, *P* = 0.029), and the CONT and TR + SUP group (mean difference = 28.53, *P* = 0.001). Additionally, significant reductions were observed between the TR and SUP, and TR groups (mean difference = 13.17, *P* = 0.010) as well as between the SUP, and TR groups (mean difference = 16.81, *P* = 0.001) (Fig. 5).

## Discussion

The present study aimed to investigate the combined effect of aerobic exercise and NBS superfood supplementation on some ferroptosis associated biomarker in methadone-dependent men. The results revealed significant time × group interactions for GPX4, SLC7A11, and ferritin levels, whereas hepcidin levels showed no significant changes. The most noticeable changes were generally observed in the combined exercise + supplement group. One of the main findings was the increase in serum GPX4 levels in the exercise-containing groups.

Increasing antioxidant capacity is one of the main approaches to inhibit ferroptosis^26^. From the perspective of exercise-induced adaptations, regular aerobic exercise has been reported to activate the Nrf2/ARE signaling pathway, which plays a role in regulating the expression of antioxidant genes^27^. This activation has been linked to increased GPX4 expression in some studies, which may be associated with reduced lipid peroxidation in cell membranes^28^. The exact mechanism in humans remains incompletely understood, but phosphorylation of protein kinases such as PKC and MAPK has been proposed as a possible pathway leading to Nrf2 nuclear accumulation and binding to antioxidant response elements (ARE) in the GPX4 gene promote^29^. A study by Liu et al. (2022) suggested that increased GPX4 activity caused by exercise may increase the resistance of cells to oxidative stress^30^. This observation could be relevant in the methadone user population, who often suffer from increased oxidative stress. On the other hand, the NBS superfood supplement, containing antioxidant compounds such as vitamin E and selenium found in wheat, may contribute to reducing oxidative stress caused by iron through effects on the SLC7A11-GPX4 pathway. SLC7A11 prevents ferroptosis by facilitating cysteine ​​uptake and increasing glutathione (GSH) synthesis, and GPX4 by neutralizing lipid peroxides^31^. Vitamin E in NBS supplements acts as a lipophilic antioxidant that may help prevent cell membrane peroxidation^32^. Sulforaphane in this supplement has been shown in preclinical models to induce the Nrf2 pathway and thereby potentially increase GPX4 synthesis. Polyphenolic compounds such as quercetin and resveratrol found in wheat may also support antioxidant activity by neutralizing free radicals^33^. Therefore, NBS supplements may offer a protective role in the pathogenesis of some diseases, including addictive disorders and diseases associated with high iron levels^34^.

The observed changes in GPX4 levels in the combined intervention group were larger than in the individual interventions, raising the possibility of an additive or synergistic effect. A review article by Zhang et al. (2024) has shown that high GPX4 levels are associated with a reduced risk of cardiovascular disease and neurodegenerative disorders^35^. These data highlight the potential value of combining exercise and targeted nutritional interventions for managing oxidative stress, although direct clinical benefits remain to be demonstrated.

The present study also observed a reduction in SLC7A11 expression, particularly in the exercise groups. Regular physical activity, by creating controlled oxidative stress, may negatively regulate xc-system-dependent pathways. Moderate-to-vigorous aerobic exercise has been reported to reduce SLC7A11 expression, possibly due to a decrease in the cell’s need to import cysteine for glutathione synthesis^36^. This adaptation requires further investigation in human populations Lee et al. (2024) showed that aerobic exercise reduces iron overload-induced ferroptosis in the prefrontal cortex of rats via the Xc^−^/GPx4 pathway^37^.

On the other hand, the superfood supplement NBS, containing active compounds such as sulforaphane and polyphenols, may have influenced this pathway. Sulforaphane has been shown in some models to regulate SLC7A11 expression by inhibiting the Nrf2 pathway. The polyphenolic compounds in this supplement may also reduce the cell’s need for the xc^−^system by directly neutralizing free radicals^25^. This mechanism explains why the reduction in SLC7A11 was associated with increased antioxidant activity in this study. This delicate balance between the reduction in SLC7A11 and the increase in GPX4 may be a smart cellular strategy to selectively sensitize damaged cells to ferroptosis, while protecting healthy cells by enhancing the GPX4 system^38^. These findings are important because they suggest that a combined exercise and supplementation intervention can lead to a smart adaptive response in the cell’s antioxidant system. The reduction in SLC7A11 may predispose inflammatory cells to ferroptosis, while the increase in GPX4 protects healthy cells from cell death^39^. This dual effect could have important implications for the design of treatment interventions in specific populations, such as methadone users.

In the present study, hepcidin levels, the main hormone regulating iron metabolism, did not change significantly. This finding is consistent with the report by Dominguez et al. (2014), which showed that continuous high-intensity exercise, despite producing significant activity of the sympathetic-adrenal system, did not lead to an increase in serum hepcidin concentrations as an acute response^40^. The lack of significant change in hepcidin after the intervention may indicate that the effects of exercise and NBS supplementation on iron metabolism were largely independent of the hepcidin pathway or that the 8-week duration was insufficient to elicit detectable changes in this hormone These results are in contrast to some studies that have reported that exercise can affect hepcidin levels. Among them, the results of a review article by Larsuphrom et al. (2021) stated that endurance exercise causes a significant increase in serum hepcidin and IL-6 levels in individuals^41^. Liu et al. (2025) examined the effects of exercise on inflammatory status, hepcidin, and markers of iron metabolism in a meta-analysis. The results showed that after acute exercise and endurance exercise, hepcidin and IL-6 concentrations increased, and markers of iron metabolism increased immediately after exercise, but returned to baseline levels or even significantly lower than baseline levels 3 h later^42^.

Several factors contribute to exercise-induced alterations in hepcidin levels, one of which is blood ferritin concentration. The current study observed a decline in blood ferritin levels following exercise. Prior research suggests a positive correlation between elevated ferritin levels and increased hepcidin expression^40^. Additionally, testosterone plays a role in modulating hepcidin; resistance training, for instance, has been shown to raise testosterone levels, thereby significantly suppressing hepcidin^43^. In contrast, aerobic exercise typically induces smaller fluctuations in testosterone compared to resistance training^44^. Given the exercise protocol employed in this study, it is plausible that the intervention did not elicit substantial changes in testosterone levels among the male participants, potentially explaining the absence of significant variations in hepcidin.

The present study observed a reduction in ferritin levels after a combined intervention of aerobic exercise and NBS superfood supplementation. Tan et al. (2023) have shown that aerobic exercise can modulate ferritin levels by reducing inflammation^45^. Regular aerobic exercise can modulate ferritin levels by lowering inflammatory markers such as interleukin-6 (IL-6)^46^. Nash et al. (2023) have shown that a single acute aerobic exercise session can lead to a temporary increase in IL-6, but in the long term, regular exercise reduces systemic inflammatory markers. From an exercise physiology perspective, aerobic exercise may reduce ferritin levels as an acute-phase protein by modulating proinflammatory cytokines such as IL-6 and TNF-α^47^. From a metabolic perspective, aerobic exercise has a direct effect on the regulation of iron homeostasis by improving insulin sensitivity and reducing insulin resistance. Pathophysiological studies have shown that hyperinsulinemia is associated with increased intestinal iron absorption and tissue ferritin accumulation^48^. At the cellular level, aerobic exercise may reduce ferritin stores by increasing the functional iron requirement for hemoglobin and myoglobin synthesis^49^.

**Fig. 6:**
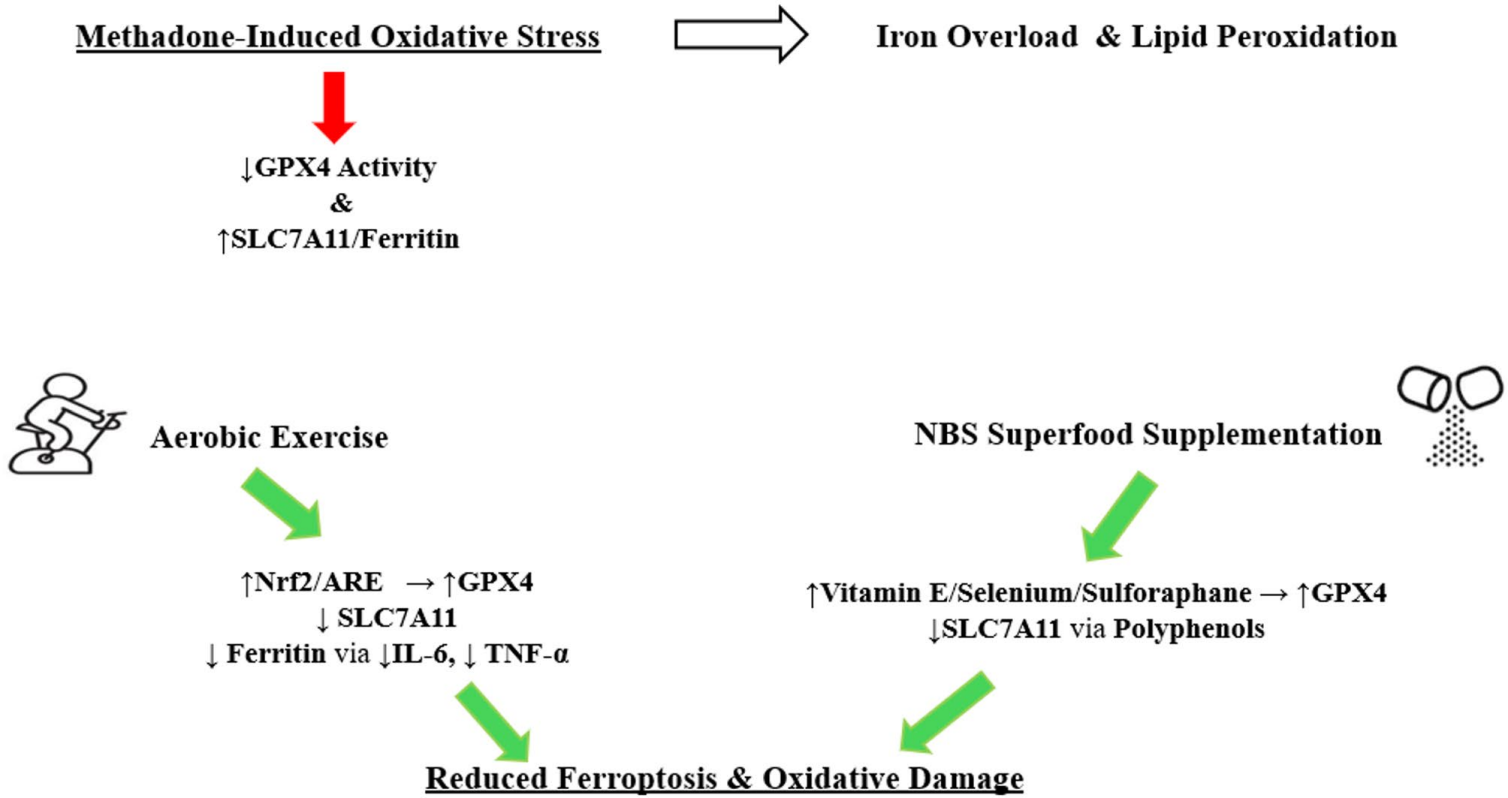
Proposed cellular pathway illustrating how aerobic exercise and NBS supplementation counteract methadone-induced ferroptosis by enhancing GPX4 activity, reducing SLC7A11 and ferritin, and modulating inflammation.

In the meantime, the superfood supplement NBS, with its polyphenolic and flavonoid compounds, may enhance the anti-inflammatory effects of exercise by inhibiting the NF-κB pathway^24^. The reduction in ferritin observed in this study most likely reflects an improvement in systemic inflammation rather than a true depletion of body iron stores. An important point in interpreting these findings is the dependence of the ferritin response on exercise parameters. While moderate-intensity exercise typically has a ferritin-lowering effect, prolonged, very intense activity may result in a transient increase in ferritin levels due to the induction of oxidative stress and muscle damage^50^. In the present study, the observed reduction in ferritin levels may be explained by the combined effects of aerobic exercise on systemic inflammation, insulin sensitivity, and iron metabolism, all of which can be explained by scientifically documented mechanisms. The anti-inflammatory compounds in wheat found in NBS supplements (such as polyphenols) may contribute to lowering ferritin levels by reducing IL-6. Although wheat does not directly affect Hepcidin, it may may indirectly reduce the need for increased Hepcidin by reducing systemic inflammation^51^. The proposed molecular mechanism underlying these changes is illustrated in Fig. 6, which highlights the interplay between increased GPX4 activity, reduced SLC7A11 and ferritin, and the protective roles of aerobic exercise and NBS supplementation against methadone-induced oxidative stress.

## Conclusion

The findings of this study indicate that the combination of aerobic exercise and NBS supplementation can modulate blood markers associated with iron metabolism and antioxidant status in methadone-treated addicts. These patients are susceptible to cellular damage due to altered iron homeostasis and potential oxidative imbalances. Our findings indicate that this combined intervention may support antioxidant defenses through increased GPX4 enzyme activity and improved SLC7A11 pathway function, which could be facilitated by selenium, vitamin E, and polyphenols in NBS supplementation. On the other hand, aerobic exercise, by creating metabolic adaptations and potentially reducing systemic oxidative burden, may contribute to optimal conditions for maintaining cellular integrity. These combined effects could have potential protective roles against methadone-induced cellular stress and could be proposed as a supportive strategy alongside standard treatments to improve clinical outcomes in this population. However, to generalize these results, further studies with longer intervention periods and in different clinical populations are necessary.

This study has several limitations. First, we relied on indirect biomarkers of ferroptosis and oxidative stress rather than direct measurements (e.g., lipid peroxidation products such as malondialdehyde or 4-hydroxynonenal, GPX4 protein expression, or iron-dependent cell death assays). Second, the relatively small sample size (*n* = 60) and short intervention duration (8 weeks) may limit the generalizability of the findings. Third, the study included only male participants, which precludes extrapolation to females. Fourth, due to resource limitations, we could not perform detailed serum phytochemical analysis of the NBS superfood mixture to identify the specific bioactive compounds responsible for the observed effects. Future studies should incorporate direct ferroptosis assays, larger and gender-balanced cohorts, longer intervention periods, and comprehensive characterization of the supplement’s active constituents to confirm and extend these preliminary findings.

Despite these limitations, the present findings suggest that moderate-intensity aerobic exercise combined with NBS superfood supplementation may serve as a safe, complementary strategy to improve metabolic and inflammatory profiles in methadone-maintained patients. Clinicians may consider recommending such lifestyle interventions alongside standard treatment, provided they are individualized and medically supervised.

## Data Availability

The data supporting the findings of this study are available within the article. This data can be provided by the corresponding author upon reasonable request.

## References

[1] Nawi, A. M. et al. Risk and protective factors of drug abuse among adolescents: a systematic review. *BMC Public. Health*. **21** (1), 2088 (2021). 34774013 10.1186/s12889-021-11906-2 PMC8590764

[2] (UNODC). UNOoDaC. World Drug Report 2024. Vienna:. (2024). Available from: https://www.unodc.org/unodc/data-and-analysis/world-drug-report-2024.html

[3] Rass, O. et al. A randomized controlled trial of the effects of working memory training in methadone maintenance patients. *Drug Alcohol Depend.* **156**, 38–46 (2015). 26404954 10.1016/j.drugalcdep.2015.08.012 PMC4633307

[4] Halcrow, P. W. et al. Mu opioid receptor-mediated release of endolysosome iron increases levels of mitochondrial iron, reactive oxygen species, and cell death. *NeuroImmune Pharmacol. Ther.* **2** (1), 19–35 (2023). 10.1515/nipt-2022-0013 PMC10070011 37027339

[5] Zhang, Y., Shi, C., Zhang, D., Zhang, L. & Wang, L. Gong Z-j. Sulforaphane, an NRF2 agonist, alleviates ferroptosis in acute liver failure by regulating HDAC6 activity. *J. Integr. Med.* **21** (5), 464–473 (2023). 37620223 10.1016/j.joim.2023.08.002

[6] Xie, Y. et al. Ferroptosis: process and function. *Cell. Death Differ.* **23** (3), 369–379 (2016). 26794443 10.1038/cdd.2015.158 PMC5072448

[7] Li, F. J. et al. System X(c) (-)/GSH/GPX4 axis: an important antioxidant system for the ferroptosis in drug-resistant solid tumor therapy. *Front. Pharmacol.* **13**, 910292 (2022). 36105219 10.3389/fphar.2022.910292 PMC9465090

[8] Su, Z., Liu, Y., Wang, L. & Gu, W. Regulation of SLC7A11 as an unconventional checkpoint in tumorigenesis through ferroptosis. *Genes Dis.* **12** (1), 101254 (2025). 39569390 10.1016/j.gendis.2024.101254 PMC11577153

[9] Kosari, K. et al. Methadone and the kidney: dissecting gender differences in inflammation and oxidative stress responses. *Addict. Health*. **17**, 1625 (2025). 40666081 10.34172/ahj.1625 PMC12260921

[10] Zeng, X., Li, J., Yang, F. & Xia, R. The effect of narcotics on ferroptosis-related molecular mechanisms and signalling pathways. *Front. Pharmacol.* **13**, (2022). 10.3389/fphar.2022.1020447 PMC9606818 36313359

[11] Anderson, G. J. & Frazer, D. M. Current Understanding of iron homeostasis†‡. *Am. J. Clin. Nutr.* **106**, 1559S–66S (2017). 29070551 10.3945/ajcn.117.155804 PMC5701707

[12] Domínguez, R. et al. Effects of an acute exercise bout on serum Hepcidin levels. *Nutrients* **10** (2), 209 (2018). 29443922 10.3390/nu10020209 PMC5852785

[13] Xiang, Y-Y., Baek, K-W., Won, J-H., Park, Y. & Kim, J-S. Effects of lifelong aerobic exercise on Ferroptosis-Related gene expressions in kidney of aged mice. *Exerc. Sci.* **32**, 410–418 (2023).

[14] Šmid, A. N. et al. Effects of oral iron supplementation on blood iron status in athletes: A systematic Review, Meta-Analysis and Meta-Regression of randomized controlled Trials. *Sports medicine (Auckland, NZ)*. **54**(5), 1231–1247 (2024). 10.1007/s40279-024-01992-8 PMC11127818 38407751

[15] Liu, R. H. Dietary bioactive compounds and their health implications. *J. Food Sci.* **78** (s1), A18–A25 (2013). 23789932 10.1111/1750-3841.12101

[16] Azizi Jalilian, F. et al. The effects of nutrition bio-shield superfood powder on immune system function: A clinical trial study among patients with COVID-19. *Front. Immunol.* **13**. (2022). 10.3389/fimmu.2022.919402 PMC9458072 36091037

[17] Fernández-Lázaro, D. et al. The role of selenium mineral trace element in exercise: antioxidant defense system, muscle performance, hormone response, and athletic performance. A systematic review. *Nutrients* **12**(6), 1790 (2020). 10.3390/nu12061790 PMC7353379 32560188

[18] Siraj, N. Wheat germ oil: a comprehensive review. *Food Sci. Technol.* **42**, e113721 (2022).

[19] Piskin, E., Cianciosi, D., Gulec, S., Tomas, M. & Capanoglu, E. Iron absorption: Factors, Limitations, and improvement methods. *ACS Omega*. **7** (24), 20441–20456 (2022). 35755397 10.1021/acsomega.2c01833 PMC9219084

[20] Kordi, N., Azizi, M., Samadi, M. & Tahmasebi, W. The effect of aerobic training and NBS superfood supplementation on apoptosis and cardiac damage in methamphetamine-withdrawn rats. *Sci. Rep.* **15** (1), 37132 (2025). 41131035 10.1038/s41598-025-21130-2 PMC12549946

[21] Alsherify, S. M., Hassanabadi, A., Zerehdaran, S. & Nassiri Moghaddam, H. The effect of herbal product (NBS Superfood) supplementation on egg quality traits in commercial laying hens. *Poult. Sci. J.* **10** (2), 251–267 (2022).

[22] Waer, F. B. et al. Caffeine optimizes Zumba training benefits on functional performances in middle-aged women: a randomized trial study. *Sci. Rep.* **14** (1), 25657 (2024). 39463442 10.1038/s41598-024-76650-0 PMC11514171

[23] Shafiee, N. et al. Cardiac rehabilitation in coronary artery bypass grafting patients: effect of eight weeks of moderate-intensity continuous training versus high-intensity interval training. *Clin. Hemorheol Microcirc*. **83** (3), 305–314 (2023). 36683497 10.3233/CH-221605

[24] Shirinbayan, M. M., Azizi, M. & Amiri, E. The effect of one-week spinach and NBS superfood supplementation on interleukin-6, superoxide dismutase, and malondialdehyde levels after repeated bouts of wingate test in trained men. *Nutr. Metabolism*. **22** (1), 18 (2025). 10.1186/s12986-025-00911-6 PMC11866640 40012033

[25] Bayat, A., Khalkhali, A. & Mahjoub, A. Nutrition-Bio-Shield-Superfood-Healthy-and-Live-Herbal-Supplement-for-Immune-System-Enhancement. **15**, 6–9 (2021).

[26] Xiang, Y-Y., Baek, K-W., Won, J-H., Park, Y. & Kim, J-S. Effects of lifelong aerobic exercise on Ferroptosis-Related gene expressions in kidney of aged mice. *Exerc. Sci.* **32** (4), 410–418 (2023).

[27] Vargas-Mendoza, N. et al. Antioxidant and adaptative response mediated by Nrf2 during physical exercise. *Antioxidants* **8** (6), 196 (2019). 31242588 10.3390/antiox8060196 PMC6617290

[28] Kozakowska, M., Pietraszek-Gremplewicz, K., Jozkowicz, A. & Dulak, J. The role of oxidative stress in skeletal muscle injury and regeneration: focus on antioxidant enzymes. *J. Muscle Res. Cell Motil.* **36** (6), 377–393 (2015). 26728750 10.1007/s10974-015-9438-9 PMC4762917

[29] Ngo, V. & Duennwald, M. L. Nrf2 and oxidative stress: a general overview of mechanisms and implications in human disease. *Antioxid* **11**(12), 2345 (2022). 10.3390/antiox11122345 PMC9774434 36552553

[30] Liu, T. et al. Treadmill Training Reduces Cerebral Ischemia‐Reperfusion Injury by Inhibiting Ferroptosis through Activation of SLC7A11/GPX4. *Oxid. Med. Cell Longev.* **2022**(1), 8693664 (2022). 10.1155/2022/8693664 PMC9192201 35707270

[31] Stockwell, B. R. et al. Ferroptosis: A regulated cell death nexus linking Metabolism, redox Biology, and disease. *Cell* **171** (2), 273–285 (2017). 28985560 10.1016/j.cell.2017.09.021 PMC5685180

[32] Lesjak, M., Simin, N. & Srai, S. K. S. *Can. Polyphenols Inhibit Ferroptosis? Antioxid.* ;**11**(1):150. (2022). 10.3390/antiox11010150 PMC8772735 35052654

[33] Zhang, M. et al. Recent advances in the Inhibition of membrane lipid peroxidation by food-borne plant polyphenols via the nrf2/gpx4 pathway. *J. Agric. Food Chem.* **72** (22), 12340–12355 (2024). 38776233 10.1021/acs.jafc.4c00523

[34] Guo, C., Chen, L. & Wang, Y. Substance abuse and neurodegenerative diseases: focus on ferroptosis. *Arch. Toxicol.* **97** (6), 1519–1528 (2023). 37100932 10.1007/s00204-023-03505-4

[35] Zhang, W., Liu, Y., Liao, Y., Zhu, C. & Zou, Z. GPX4, ferroptosis, and diseases. *Biomed. Pharmacother.* **174**, 116512 (2024). 38574617 10.1016/j.biopha.2024.116512

[36] Liu, H. et al. Aerobic exercise alleviates doxorubicin-induced cardiotoxicity via inhibition of ferroptosis. *Chemotherapy* **70 **(3), 137–152 (2025). 10.1159/000546096 40349681

[37] Li, C. et al. 8-weeks aerobic exercise ameliorates cognitive deficit and mitigates ferroptosis triggered by iron overload in the prefrontal cortex of APP Swe/PSEN 1dE9 mice through Xc−/GPx4 pathway. *Front. Neurosci.* **18**, 1453582 (2024). 10.3389/fnins.2024.1453582 PMC11417105 39315073

[38] He, R. et al. α-Ketoglutarate alleviates osteoarthritis by inhibiting ferroptosis via the ETV4/SLC7A11/GPX4 signaling pathway. *Cell. Mol. Biol. Lett.* **29** (1), 88 (2024). 38877424 10.1186/s11658-024-00605-6 PMC11177415

[39] Sheng, W., Liao, S., Wang, D., Liu, P. & Zeng, H. The role of ferroptosis in osteoarthritis: progress and prospects. *Biochem. Biophys. Res. Commun.* **733**, 150683 (2024). 39293333 10.1016/j.bbrc.2024.150683

[40] Domínguez, R., Vicente-Campos, D. & Lopez Chicharro, J. Hepcidin response to exercise: A review. *Turkish J. Endocrinol. Metabolism*. **18**, 84–91 (2014).

[41] Larsuphrom, P. & Latunde-Dada, G. O. Association of serum hepcidin levels with aerobic and resistance exercise: a systematic review. *Nutrients* **13**(2), 393 (2021). 10.3390/nu13020393 PMC7911648 33513924

[42] Liu, S. et al. *Effects of Exercise on the Hepcidin Inflammatory Status and Markers of Iron Metabolism: A meta-analysis* (Science & Sports, 2025).

[43] Goto, K. et al. Resistance exercise causes greater serum Hepcidin elevation than endurance (cycling) exercise. *PLoS One*. **15** (2), e0228766 (2020). 32106271 10.1371/journal.pone.0228766 PMC7046260

[44] Kumagai, H. et al. Vigorous physical activity is associated with regular aerobic Exercise-Induced increased serum testosterone levels in Overweight/Obese men. *Hormone Metabolic Res. = Hormon- Und Stoffwechselforschung = Horm. Et Metab.* **50** (1), 73–79 (2018). 10.1055/s-0043-117497 28934816

[45] Tan, L. et al. Effect of exercise on inflammatory markers in postmenopausal women with overweight and obesity: A systematic review and meta-analysis. *Exp. Gerontol.* **183**, 112310 (2023). 37844768 10.1016/j.exger.2023.112310

[46] Wang, Y. H. et al. Effects of aerobic exercise on inflammatory factors in healthy adults: a meta-analysis. *Eur. Rev. Med. Pharmacol. Sci.* **26**(12), 4163–4175 (2022). 10.26355/eurrev_202206_29053 35776016

[47] Nash, D. et al. IL-6 signaling in acute exercise and chronic training: potential consequences for health and athletic performance. *Scand. J. Med. Sci. Sports*. **33** (1), 4–19 (2023). 36168944 10.1111/sms.14241 PMC10092579

[48] Zhang, T., Liu, Y., Yang, Y., Luo, J. & Hao, C. The effect and mechanism of regular exercise on improving insulin impedance: based on the perspective of cellular and molecular levels. *Int. J. Mol. Sci.* **26** (9), 4199 (2025). 40362436 10.3390/ijms26094199 PMC12071773

[49] Solberg, A. & Reikvam, H. Iron status and physical performance in athletes. *Life (Basel Switzerland)*. **13**(10), 2007 (2023). 10.3390/life13102007 PMC10608302 37895389

[50] heidarianpour, A., Aghamihammadi, M. & Keshvari, M. The effect of different exercise training modes on serum ferritin and iron levels of type 2 diabetic rats. *Pathobiology Res.* **25** (2), 22–29 (2022).

[51] Charlebois, E. & Pantopoulos, K. Iron overload inhibits BMP/SMAD and IL-6/STAT3 signaling to Hepcidin in cultured hepatocytes. *PLoS One*. **16** (6), e0253475 (2021). 34161397 10.1371/journal.pone.0253475 PMC8221488

